# Esophageal Granular Cell Tumor in a 28-year-old: A Unique Cause for Dysphagia

**DOI:** 10.7759/cureus.2679

**Published:** 2018-05-23

**Authors:** Vincent M Pronesti, Kanika Goel, Marcia Mitre

**Affiliations:** 1 Internal Medicine, Allegheny Health Network, Pittsburgh, USA; 2 Department of Pathology, Allegheny Health Network, Pittsburgh, USA; 3 Department of Gastroenterology, Allegheny Health Network, Pittsburgh, USA

**Keywords:** granular cell tumor, dysphagia, esophagus, esophagogastroduodenoscopy, endoscopic ultrasound, esophageal manometry

## Abstract

This is the case of a 28-year-old female who presented with a complaint of dysphagia and was diagnosed with the rare disease of esophageal granular cell tumor (GCT) after esophagogastroduodenoscopy (EGD) and endoscopic ultrasound (EUS). The case acknowledges the wisdom of maintaining a broad differential for a common complaint. It also serves to reiterate the clinical and pathologic criteria for the diagnosis of a granular cell tumor of the esophagus.

## Introduction

Granular cell tumors (GCTs) are usually found in the skin over 70% of the time but can be found in the gastrointestinal tract 8% of the time, with the most common site being the esophagus [1-3]. Of all esophageal tumors, only 1% are GCTs [1,4]. GCTs are exceedingly rare and there are only approximately 300 reported cases in the literature since their discovery in 1931 [5-6]. This is the case of a healthy 28-year-old female who presented with dysphagia and was found to have an esophageal granular cell tumor on esophagogastroduodenoscopy (EGD) and endoscopic ultrasound (EUS). Biopsies were conducted, with pathology confirming the diagnosis of a granular cell tumor. Many prior studies have shown that the typical age of presentation for GCT is between the fourth and sixth decades of life, making this case unique, as the patient was relatively young [7-8]. This report also displays the hallmarks of this disease and gives a comprehensive overview of diagnostic characteristics. The case was first presented in a much-simplified poster format at the 2018 Society of General Internal Medicine Annual Meeting. This greatly expanded work seeks to accomplish the goal of reminding clinicians to remain cognizant of an unusual disease presenting in a common manner.

## Case presentation

A 28-year-old African American female with no significant past medical or surgical history presented with complaints of dysphagia to liquids, globus sensation, frequent throat clearing, and cough for approximately the past two to three years. She also had intermittent regurgitation without blood or undigested food. A review of systems was otherwise negative for weight loss, odynophagia, dyspepsia, reflux, post-prandial bloating, early satiety, shortness of breath, recent illness, fevers, and chills. She had no sick contacts. Family history was non-contributory. The patient's social history included current cigarette smoking of 0.5 pack per day for the past one to two years, daily smoking of marijuana, and no alcohol consumption. The patient was not taking any medications. She had undergone a barium swallow within the past two years, with no pathologic findings. For these symptoms, she underwent an initial esophagogastroduodenoscopy with the findings of a white sub-mucosal lesion in the mid-esophagus at 35 cm. Biopsies were not taken of the mass at that time, but there were biopsies of the proximal and distal esophagus surrounding the lesion, with pathology indicating benign mucosa with no eosinophilic infiltrate. Esophageal, gastric, and duodenal mucosa was otherwise unremarkable. She was referred to a gastroenterologist at our institution for a follow-up endoscopic ultrasound.

**Figure 1:**
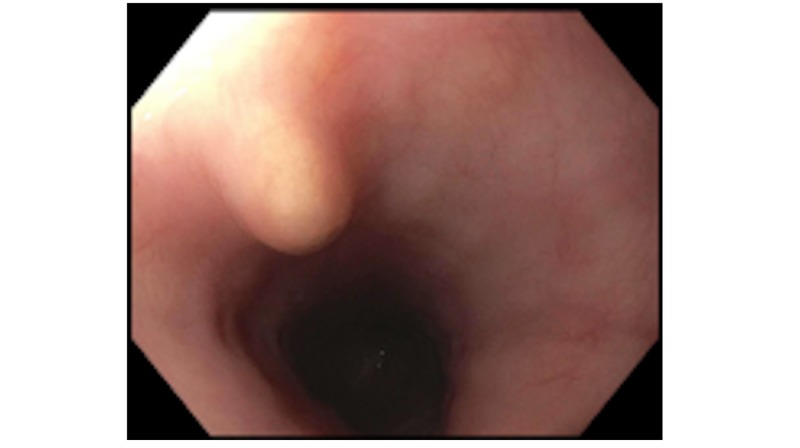
Intact granular cell tumor White sub-mucosal lesion at 35 cm from the incisors in the mid-esophagus.

**Figure 2:**
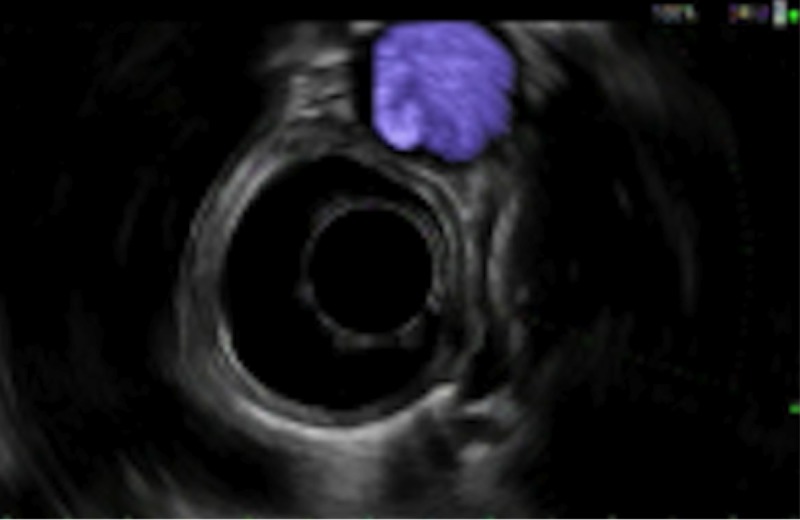
Sonographic image of the nodule on EUS Intramural, sub-epithelial nodule located within the deep mucosa of layer 2 on endoscopic ultrasound (EUS).

**Figure 3:**
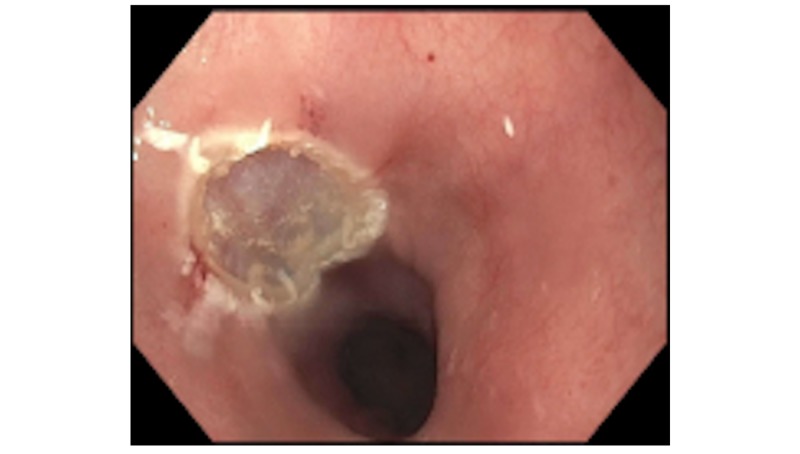
Site of the nodule after snare mucosal resection The nodule was removed with band ligation followed by snare mucosal resection.

EUS was conducted and showed a single 4-mm sub-mucosal nodule in the middle third of the esophagus at 35 cm from the incisors. The nodule was yellow and firm with a negative pillow sign. This is displayed in Figure 1. Sonographically, the lesion was oval and described as intramural, sub-epithelial, anechoic, and within the deep mucosa in layer 2 with well-defined borders. This image is seen in Figure 2. Saline was injected to raise the lesion and band ligation with snare mucosal resection was performed. The result is shown in Figure 3. The pathology of the tumor revealed a granular cell tumor with peripheral and deep margins negative. She was referred to medical oncology for further work-up.

Medical oncology reassured the patient that a granular cell tumor is typically a benign mucosal tumor with a low risk of recurrence and malignant degeneration. It was recommended that she continue to follow with gastroenterology for surveillance endoscopy. She was also given a referral for an esophageal motility specialist due to her continued dysphagia. She did have subsequent esophageal manometry conducted, which showed 60% peristalsis, indicating a degree of impaired peristalsis, along with the presence of a manometric hiatal hernia. She continues to follow with her primary care physician and gastroenterologist for further management but continues to have a degree of dysphagia.

## Discussion

Granular cell tumors are rare tumors that are usually found in the skin and subcutaneous tissues, together comprising approximately 70% of total disease incidence [1-2]. GCTs can be found in the gastrointestinal tract 8% of the time, with the most common site being the esophagus [1,3]. Of all esophageal tumors, only 1% are GCTs [1,4]. Since the first reported case of esophageal GCT in 1931, there have been only approximately 300 cases discussed in the literature [5-6]. The disease entity was first reported by Abriksoosoff in 1926 as an oral lesion, with a later discovery of the first esophageal presentation in 1931 [7]. The neoplasm arises from neural or Schwann cell origin [8]. This case is unique because the patient presented at an atypical young age with an otherwise typical presentation of GCT. Although GCT can occur at any age, it is more common between the fourth and sixth decades of life [8]. Our patient was only 28 years old and had been having the symptoms for which she presented for two to three years. GCT is more common in women than men by a ratio of 2-3:1 [8]. It is also more common in African American than white patients in a 3:1 ratio [8]. These characteristics of gender and race align with our patient’s demographic. GCTs are often asymptomatic, but the most common presenting symptom is dysphagia when the esophagus is involved [1].

It is important to note that this patient’s lesion followed the general rules for presentation, tumor location, and characteristics for GCT. One of the most common symptoms on presentation is indolent dysphagia with few other symptoms. Endoscopically, GCTs are typically yellow-white in color, firm, with a negative pillow sign [1]. This patient’s findings on endoscopy were consistent with this presentation, and are seen in Figure 1. They are also typically hypoechoic with smooth margins arising from the second or third layer of the distal esophagus [1]. The patient’s lesion was found in the second layer within the deep mucosa of the middle third of the esophagus, as seen in Figure 3.

**Figure 4:**
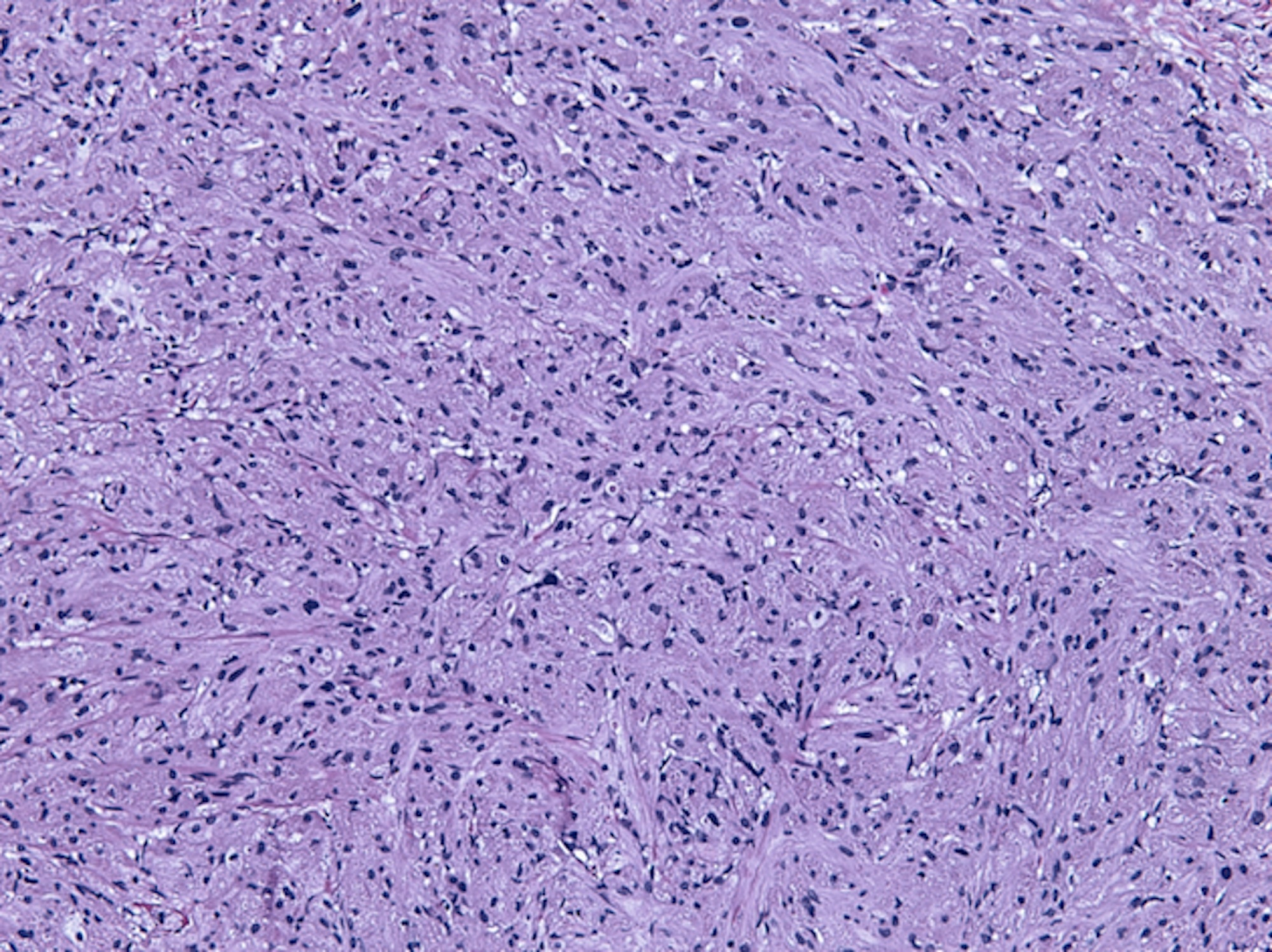
Pathologic image of a granular cell tumor On pathology, granular cell tumors have large, polygonal cells with low nuclear-cytoplasmic ratios.

It is useful to note the histologic and immunohistochemical features of GCT. Histologically, GCTs have large polygonal cells that often contain eosinophilic granules with low nuclear-cytoplasmic ratios [1,5]. These characteristics are seen in Figure 4. Our patient had identification of GCT solely on histology with all of the above characteristics, but immunohistochemical staining would identify positive S-100, PAS, neuron-specific enolase, and nestin [5]. This positive staining pattern reinforces the fact that GCT emanates from neuronal origin.

After follow-up for mucosal resection, our patient was referred to medical oncology for further evaluation. GCTs are normally benign, but there have been descriptions of malignant potential in the literature in 4% of lesions, generally with those lesions over 4 cm [1]. Fanburg-Smith identified six criteria to clarify malignant potential [1,9]. The six histologic criteria are necrosis, spindling, vesicular nuclei with large nucleoli, increased mitotic activity, high nuclear to cytoplasmic ratio, and pleomorphism [1,9]. With these criteria in mind, unresected lesions that are asymptomatic and under 1 cm should be monitored with EGD or EUS every one to two years for an increase in size [1]. If the lesions are symptomatic or over 1 cm, endoscopic mucosal resection or endoscopic sub-mucosal dissection is recommended [1]. Prior to resection, it is necessary to characterize the depth of the lesion with EUS to reduce the risk of perforation [1].

All of the findings discussed help to differentiate GCT from other entities on the differential, which would include lipoma, leiomyoma, or gastrointestinal stromal tumor (GIST). When the patient first presents to the clinician, the physician must be cognizant of the broad differential for dysphagia and that not all healthy patients have a common etiology driving the presentation. This case demonstrates the presentation typical of GCT, which is a rare cause for dysphagia. This also presents an interesting diagnostic case because although our patient did have symptoms of dysphagia, the finding of GCT was possibly incidental, as she continues to have symptoms after the resection of the GCT and has since had an esophageal manometry study with decreased peristalsis. The finding of GCT is also possibly incidental, as the patient’s mass was only found to be 4 mm and, normally, only masses over 1 cm cause symptoms [5]. These findings suggest that the GCT may not be the sole contributor to her dysphagia, but that she may also have an underlying esophageal motility pathology.

## Conclusions

This case acknowledges the wisdom of maintaining a broad differential for a common complaint of dysphagia and contributes to a clinician’s diagnostic repertoire. Although a granular cell tumor is uncommon, with approximately 300 cases being described in the literature, it was discovered in this otherwise healthy young patient. Maintaining a broad differential allows the seasoned internist to recognize that unique pathologies do occur and need appropriate intervention or referral. Other entities that exist on the differential for such a presenting complaint include gastrointestinal stromal tumor or lipoma and each requires a very different management plan.
